# Misreporting of Energy Intake Is Related to Specific Food Items in Low-Middle Income Chilean Adolescents †

**DOI:** 10.3390/children9020293

**Published:** 2022-02-21

**Authors:** Angela Martínez-Arroyo, Lais Duarte Batista, Camila Corvalán Aguilar, Regina Mara Fisberg

**Affiliations:** 1 Food Behavior Research Center (CEIC), School of Nutrition and Dietetics, Faculty of Pharmacy, University of Valparaíso, Valparaíso 2360102, Chile; angela.martinez@uv.cl; 2 Department of Nutrition, School of Public Health, University of São Paulo, Sao Paulo 01246-904, Brazil; laisduarte@usp.br; 3 Institute of Nutrition and Food Technology (INTA), University of Chile, Santiago 7830420, Chile; ccorval@gmail.com

**Keywords:** misreporting, under-reporting, over-reporting, energy intake, food items, adolescents

## Abstract

Background: Misreporting of energy intake (EI) in self-reported dietary assessment is inevitable, and even less is known about which food items are misreported by low-middle income adolescents. We evaluated the prevalence of misreporting of energy intake and its relationship with nutrients and food intake. Methods: We analyzed 24 h dietary recalls collected from 576 adolescents (52.08% boys) from southeastern Santiago. Anthropometrics measurements and information about sociodemographic characteristics were obtained during clinical visits. The method proposed by McCrory et al. was used to identify under-reporters (UnRs), over-reporters (OvRs), or plausible reporters (PRs). Food items were collapsed into 28 categories and every food item was expressed as a percentage of total EI. Logistic regression models were performed to investigate the factors associated with misreporting, and a two-part model was used to estimate the difference in the percentage of EI between UnRs versus PRs, and OvRs versus PRs in each food item. Results: Half of the participants were classified as UnRs and 9% were OvRs. UnR was higher among boys (62%) and adolescents with overweight and obesity (72%). OvR was higher among adolescents with normal weight. UnRs had a lower intake of energy from cookies/cake, chocolate/confectionery, and a higher intake of vegetables and eggs than PRs. OvRs had a higher intake of cookies/cake, chocolate/confectionery, and a lower intake of fruit, white milk, and yogurt than PRs. Conclusions: A high frequency of UnR among boys and participants with excess weight was found in this study. Healthy and unhealthy foods are reported differently between UnRs and OvRs of energy intake, indicating that bias is specific for some food items that adolescents commonly eat.

## 1. Introduction

Self-reported dietary surveys are important instruments to assess and monitor food and nutrition programs and public health policies. However, the misreporting (under- and over-reporting) of energy intake (EI) is a major limitation in the collection of self-reported dietary intake [1].

Misreporting of energy, which is defined as reported energy intake below or above the true intake, is a systematic bias that can lead to incorrect results and conclusions in dietary studies [1,2]. Self-reported dietary assessments, such as 24-h recall (24HR), are, therefore, prone to this bias, affecting not only the estimation of total energy intake (EI), but also the macronutrient composition of the diet [1]. Food omissions and inaccurate portion size estimates have been found to be two major contributors to this source of error [3]. However, there is less understanding about the extent to which food items and nutrients are misreported. In adolescents, this evidence is even more scarce. Previous studies have shown that, compared to plausible reporters, individuals who underreport their energy intake had higher protein but lower fat intake as a percentage of energy contribution. In contrast, over-reporters had higher fat [4,5] and lower carbohydrate intake [5]. Additionally, unhealthy foods such as soft drinks and confectionery may be under-reported when we use self-reported instruments, as opposed to healthy food, such as fruit and vegetables, which are frequently over-reported [5,6,7,8,9].

There is little evidence regarding the magnitude and factors associated with misreporting energy intake in childhood, especially from Latin America and Caribbean (LAC) countries [6,10,11]. Results from a literature review [10] concluded that misreporting children’s EI in western countries is frequent, as in adults, and stand at 2% to 85% for underreporting and 3% to 46% for over-reporting [10]. Besides, under-reporting in children is usually more prevalent than over-reporting, and it has been consistently associated with age and adiposity [4,10,12]. On the other hand, considering sex and social desirability, although some studies have shown an association with misreporting of EI, this association is not well-established in studies evaluating children and adolescents [10].

It is essential that nutritional epidemiology studies better understand which foods tend to be misreported by this age group because the reasons for misreporting can differ between adolescents and adults. Adolescents are likely to have less-structured meals and snacks and to consume foods outside the house. Besides, they have a lack of knowledge in estimating food portion size and home dish preparation methods, plus a lack of motivation during interviews. They are also more exposed to a beauty standard. Together, all these factors could impact their ability to accurately report food intake [4,8,9,10]. Considering that adolescence is a critical life stage, diet is a relevant aspect during this period, which can affect their future health conditions [13].

Identifying who and which food groups are under- or over-reported is essential to evaluate current public policies implemented in Chile, such as policies on food labels, marketing, the sugar-sweetened beverage tax on prices, and other health programs, which must be evaluated with reliable dietary and health data Thus, this study aims to examine the differences in food items and non-dietary characteristics among under-, plausible, and over-reporters of energy intake in adolescents from low–middle-income families.

## 2. Materials and Methods

### 2.1. Study Population

The study of the Growth and Obesity Chilean Cohort (GOCS) began in 2006. It includes information about 1195 children born in six counties of Santiago, Chile, between 2002 and 2003. Previously, the features of recruitment techniques and design of GOCS have been explained [14]. At the Health Clinic, participants received a physical exam of anthropometric measurements, pubertal development [15], and dietary intake via 24 h recall (24HR).

For this cross-sectional study, we used the data collected in GOCS follow-up during the years 8–9. A sample of 913 adolescents who had dietary data assessment was evaluated between 2014 and 2015. In the present study, we included all adolescents with two 24HRs who had anthropometric measurements acquired in the course of the clinic visits. We had to exclude 337 participants because they did not have a second 24HR or weight and height measurements available. Our total analytic sample was 576 adolescents.

### 2.2. Dietary Data Collection

24HR was collected by two trained dietitians using the multiple-pass method developed by USDA to help the participants capture detailed information about all foods during the previous day [16]. During the in-person interview, adolescents were accompanied by one caretaker (a parent or a guardian) who was aware of their food intake during the previous day. Details such as mealtimes, cooking methods, and food brands were also collected. We used household measures (i.e., glasses, mugs, bowls, and plates) and photographic Atlas of Chilean Foods and Typical Preparations [17] to assist the participants in reporting food portion size.

Self-reported foods and beverages informed by the 24HRs were converted into nutritional values using Nutrition Data System for Research software 2014 version (Nutrition Coordinating Center, University of Minnesota, Minneapolis, MN, USA). Food nutritional composition available in the Chilean food composition tables (TCA) and brand local food were compared to the values described in the software, correcting them if the concordance rate was not between 80% and 120% in order to achieve food harmonization. Foods and beverages consumed by at least 5% of the sample (n = 828) were collapsed into 33 food items (Supplemental Table S1).

EI was estimated in kcal/d, and macronutrient intakes (fat, protein, carbohydrate, total sugar, and saturated fat) were expressed in g/d, considering the mean of the two 24HRs. Subsequently, macronutrients were energy-adjusted using the density method. Sodium was expressed in mg by 1000 kcal/d. The energy of food items (means of the two 24HRs) was converted into a percentage of total EI.

### 2.3. Misreporting of Energy Intake

Firstly, we used Dietary Reference Intake (DRI) equations according to the age, sex, and weight status of the adolescents to estimate the predicted energy requirements (*pER*) [18]. Due to a high sedentarism level, according to the last survey of physical activity in Chilean adolescents, we used a low physical activity level [19]. To identify categories of misreporting of *EI*, we applied the method proposed by McCrory et al. [20] and updated by Huang et al. [21] according to the following equation:

±1 SD=√((CVrEI)^2/d+(CVpER)^2+(CVmTEE)^2)

where *CV* is the coefficient of variation; *rEI* is the mean reported energy intake of the two 24HRs; *d* is the number of days of dietary assessment (*d* = 2); *pER* is the predicted energy requirement; and *mTEE* is the measured total energy expenditure by doubly labeled water (Supplemental Table S2).

We calculated the coefficient of variation of *rEI* for this specific sample (*CVrEI* = 26) [22]. The error in the equations for *pER* was calculated by dividing the *SD* of the residuals in those equations by the mean total energy expenditure according to gender and age (*CVpER* = 5.5) [18], and measurement error and day-to-day biological variation in the total energy expenditure estimated by the doubly labeled water method (*CVmTEE* = 8.2) [18,23]. Therefore, the ±1 *SD* cut-offs were equal to ±21% IE, to identify adolescents as under-reporters (UnRs), plausible reporters (PRs), or over-reporters (OvRs) of EI, if the reported energy intake (*rEI*) was <1, ±1, or >1 *SD* of the predicted energy requirements, respectively.

### 2.4. Covariates

At the Health Clinic, we measured weight and height according to standardized procedures and these were collected in duplicate by two trained dietitians. We estimated BMI-for-age z-scores based on the WHO 2007 Growth Reference [24]. The term overweight was used to refer to an overweight and obesity nutritional status combined (≥1SD BMI z−scores ). Additionally, maternal BMI also was estimated to identify mothers with obesity [25], and pubertal development (breast or genitalia) was checked in girls and boys, respectively, to classify them according to Tanner stages [26]. Mothers of the adolescents self-reported their (i) highest education level, (ii) screen time (h/d) as a proxy variable of sedentary behavior (≥2 h/day or <2 h/day) [27]; (iii) hours of sleep time (h/d); and (iv) consumption of scholar meals (yes or no) obtained through the school meal program.

### 2.5. Statistical Analysis

The statistical software Stata (version 16.0, StataCorp LP, College Station, TX, USA) was used to perform the analyses with two-sided alpha = 0.05. Quantitative variables were tested for normality using the Kolmogorov–Smirnov test. Characteristics of the study sample are presented as means or medians for continuous variables and as percentages for categorical variables, according to the classification of EI (UnR, PR, OvR). Logistic regression analysis was performed to investigate the association between sociodemographic information, nutritional status, behaviors, macronutrients, and key nutrient intake with misreporting, considering UnRs and OvRs as the outcome variables (reference category: Plausible reporters).

A two-part model with log-link and gamma distributions was considered for food items [28], accounting for the probability to consume a type of food and the amount of food consumed. We estimated the difference in the percentage of energy intake between UnRs versus PRs, and OvRs versus PRs in each food item. Both probabilities and amount models were adjusted by the covariates of age, sex, and overweight (±1 SD z score BMI). For this analysis, we excluded food items that did not contribute to energy intake, for instance, water, artificial sweeteners, diet soft drinks, low-caloric juices, tea and coffee, and salad dressings (lemon, salt, vinegar).

## 3. Results

Adolescents included 300 boys (52%) and 276 girls (48%), with a mean age of 12.1 years (SD = 0.68). PRs were defined as having a ratio of energy intake to estimated energy requirement in the range of 0.79 to 1.21, UnRs were <0.79, and OvRs had a ratio >1.21. The prevalence of UnRs was 51%, and only 9% of the participants were classified as over-reporters. Girls were more accurate reporters than boys, with a proportion of 59% girls among the PRs. About half of the sample were overweight (49.5%), where three out of four adolescents were UnRs (72.2%), and PRs and OvRs were more frequent in adolescents with normal weight (71% and 90%, respectively). Adolescents whose mothers had obesity were mainly UnRs (43.8%), and on the other hand, adolescents with mothers of normal weight were PRs or OvRs (PR = 71% and OvR = 74%). More details on the general characteristics of the study population according to categories of misreporting are shown in Table 1.

Adolescents who underreported their energy intake were less likely to be girls, more likely to be overweight or obese, and have mothers with obesity. Besides, they reported higher contributions of carbohydrate intakes to EI and fewer contributions of total and saturated fat. On the other hand, adolescents who over-reported their EI were less likely to be overweight or obese, to have an advanced tanner stage, and their mothers had fewer years of education. Additionally, they reported a higher contribution of total fat intake to EI and fewer contributions of proteins (Table 2).

**Figure 1:**
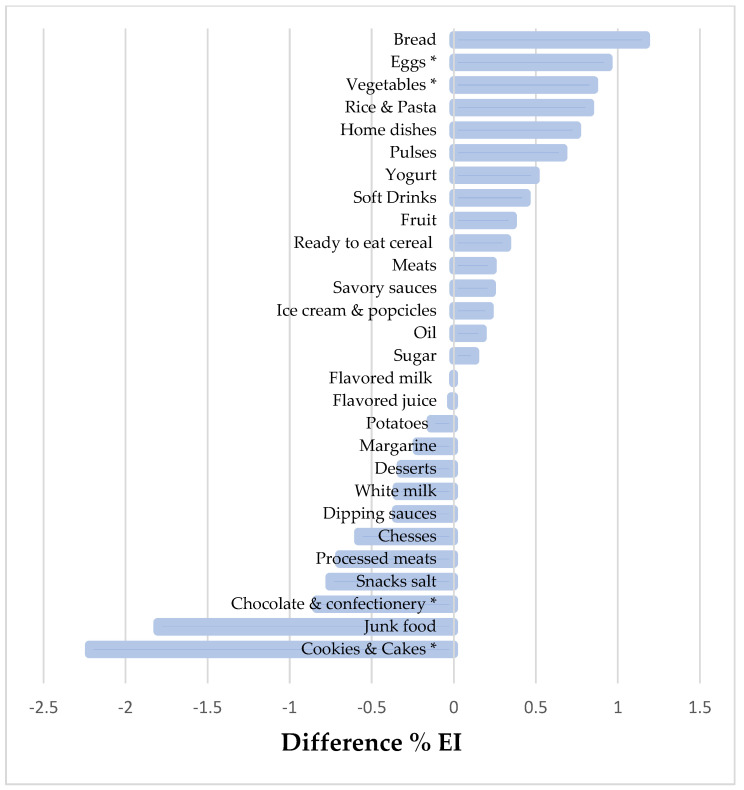
Difference % EI food items in under-reporters versus plausible reporters of energy in adolescents. Santiago, Chile, 2014–2015. Two-part model adjusted by the covariates age, sex, and overweight (±1 SD z score BMI). * *p*-value < 0.05.

**Figure 2:**
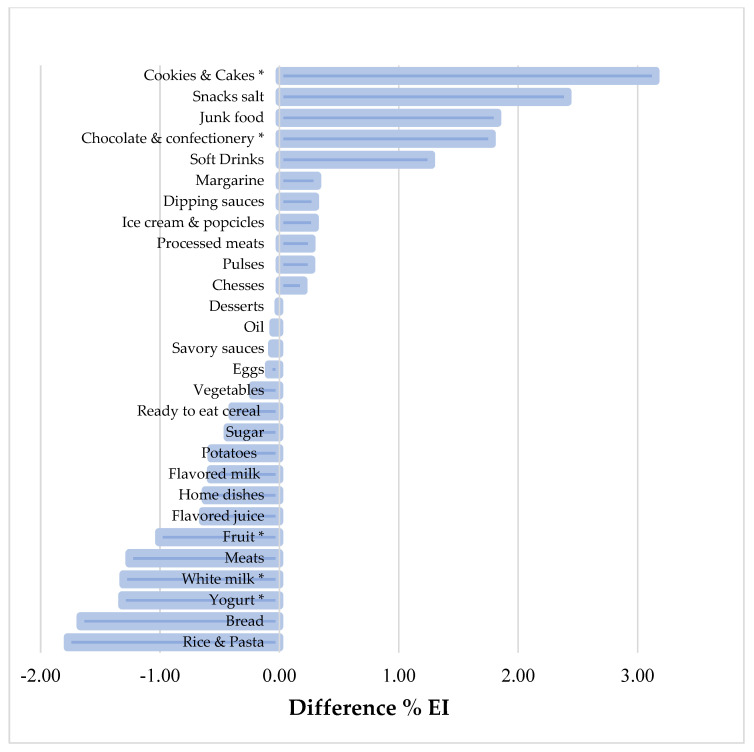
Difference in % EI of food items of over-reporters versus plausible reporters of energy in adolescents, Santiago—Chile, 2014–2015. Two-part model adjusted by the covariates age, sex, and overweight (±1 SD z score BMI). * *p*-value < 0.05.

Figure 1 and Figure 2 show the mean energy intake contribution (%) of some food items among UnRs versus PRs and OvRs versus PRs, respectively. The 28 food groups account for 95% of the total energy intake of the participants. The top five food groups that contributed most to total EI were bread (17.8%); rice and pasta (9.1%); cookies and cakes (7.8%); junk food (6%); and meats (5.4%) (Supplemental Table S3). In comparison to plausible reporters, we observed that UnR adolescents reported less EI from non-core foods such as cookies and cakes, chocolates and confectionery (*p* < 0.05), and higher reported energy contribution from core foods, such as vegetables and eggs (*p* < 0.05). Nevertheless, OvR adolescents had a higher energy contribution from cookies and cakes; chocolate and confectionery groups, and lower reported energy intake from milk, fruit, and yogurt (*p* < 0.05).

## 4. Discussion

Misreporting of energy intake was highly prevalent (frequency of UnR was higher than OvR) in this sample of Chilean adolescents from low–middle-income families. Under-reporting was more frequent in adolescents with overweight or obesity, while over-reporting was more frequent in normal-weight adolescents. Our results showed that under- and over-reporting of EI were specific for some food items, which were commonly eaten snacks (cookies and cakes, chocolate, and confectionery) between meals.

The frequency of UnRs and OvRs found in this sample of adolescents agree with the available evidence, but this varies between studies [10]. This variation could be explained by the use of different dietary instruments to assess dietary intake, equations for predicting the energy requirements, or by the criteria to estimate cutoff values to evaluate misreporting [29]. Moreover, characteristics such as socioeconomic status (SES) and possible cultural differences among the studies’ population should also be considered [6,30]. We used the method originally proposed by McCrory et al. [20] and updated by Huang et al. [21] because it is a simple approach to assess the plausibility of reported energy intake. This method considers the within-subject errors predicted by EER equations DRI [18] and *rEI*, including measurement error and normal day-to-day variation.

An intriguing finding in the current study was the higher frequency of UnRs among boys than among girls, differing from other studies [6,10]. Nevertheless, the relation between sex and the occurrence of misreporting EI during childhood and adolescence is still not conclusive. Boys could have less interest in being informed on their food intake than girls, especially during adolescence, because it is a key moment for shaping gender attitudes. Stereotypical norms or engaging in stereotypically feminine activities (e.g., household chores, such as cooking) [31] could make girls self-report their intake more accurately [32]. Studies are needed to better understand the differences that could be attributed to sex. Underreporting was associated with overweight or obesity status, similar to those reported to other children’s studies [4,8,33] and in adults [9,10]. The desire to under-report unhealthy food, consume food outside the home or at unusual times, and social desirability may contribute to the under-reporting [9,34]. Additionally, maternal obesity was also associated with UnRs, which should be considered since mothers/caregivers helped to answer the 24HRs. This could result in socially acceptable answers because they want to be seen as providing healthy foods for their children [32].

As expected, the bias in the total misreporting of EI was associated with bias in estimating macronutrient intake. Our findings were consistent with other studies in Australian [5] and European [4] children, who also under-reported lower total and saturated fat percentage and a higher intake of carbohydrates [4] and proteins as a percentage of their energy intake [4,5]. These findings should be interpreted carefully because macronutrients are part of food or meals. The differential reporting of macronutrients could be as a result of particular food types, meals, or snacks that are more likely to be misreported at this age [6,9,10]. It is important to highlight that UnR adolescents reported more core foods such as vegetables and eggs and fewer non-core or unhealthy foods such as cookies and cake and chocolate, and confectionery. These associations are consistent with previous studies in European [8], Australian [5], and Japanese children [35]. Thus, this food selection bias must be considered in dietary surveillance. For this reason, standardized procedures such as the multiple-pass method, as well as the use of household measures or a local photographic atlas must be applied by trained interviewers. During interviews, they should highlight these food items are commonly consumed by adolescents because it is not clear whether these foods are consciously under-reported, forgotten, or under-eaten [36].

On the other hand, OvRs of EI were more frequent among adolescents with normal-weight status than adolescents with overweight or obesity, consistent with previous studies [8,35,37]. Moreover, adolescents in the advanced Tanner stage had lower odds of being classified as OvRs and a low maternal education was associated with a higher probability of being an OvR. The Tanner stage could be a proxy of age in adolescents, but we did not find an association between age and OvRs. In relation to the parental level of education, studies are inconclusive, because both higher and lower parental education has been associated with UnRs but not with OvRs [4,30].

When we observed food item consumption (as % EI) of OvR adolescents, they reported a higher intake of non-core foods, such as chocolates and confectionery and cookies and cakes, and a lower intake of milk, yogurt, and fruits than PRs. Therefore, our results regarding macronutrient intake in OvR adolescents showing a higher intake of total and saturated fat and a lower intake of protein than PR are expected, as a consequence of the foods consumed. Findings were consistent with other studies in European [4,8] and Australian children [5]. Considering that non-core foods usually have a high energy density and OvRs adolescents were mainly of normal weight status, we thought that non-core food could be considered socially desirable for this sample of adolescents from families with low–middle incomes. Besides, unhealthy food and beverages have bigger marketing campaigns that could increase dietary intake and influence dietary preferences [38]. Further research is needed to understand and confirm the present findings.

Although this study presented and reinforced several insights regarding the misreporting of energy intake in adolescents, the research is not without limitations. It can be emphasized that the sample has no representativeness nation-wise. However, GOCS is representative of Chilean adolescents in Santiago from lower- and middle-income families [16,17], which is a group of the population that is not often explored in studies. Moreover, we did not assess physical activity level objectively, and we assumed a low physical activity level for our participants [20], which might be a conservative estimation. Although, we believe this is unlikely given the extent of sedentarism in the Chilean population. As for strengths, we can mention the standard and validated protocols for anthropometric measurements and dietary assessment, reducing measurement errors or response bias. The use of the local photographic atlas [17] can also help with the accuracy of portion size estimation. Although the literature suggests that from the age of 10 children can carry out the interview on their own without the help of their parents, we recommend the help of parents to have greater precision in the reporting of culinary preparations. Finally, another strength of our study was calculating the specific CV_EI_ for our sample, as it was suggested by Black [22].

## 5. Conclusions

In this sample of Chilean adolescents, we observed a high prevalence of misreporting of EI, especially UnRs, among males, reflecting boys’ tendency to have less control or motivation in self-reporting dietary behaviors compared to girls. Weight status in adolescents was the main predictor of misreporting energy intake. Besides, we showed that under- and over-reporting of EI were specific for some food items that are commonly eaten by adolescents. Because self-reported dietary instruments are the main tool for evaluating public health nutrition policies, this selective bias should always be considered.

## Figures and Tables

**Table 1:** Characteristics of the participants in the Growth and Obesity Cohort Study 2014–2015.

	Total		UnR		PR		OvR	
	*n*	%	*n*	%	*n*	%	*n*	%
	576	100	295	51.22	231	40.1	50	8.68
***Sociodemographic***								
Age, years (mean, SD)	12.10	0.68	12.19	0.66	11.98	0.65	12.08	0.82
Sex								
boys	300	52.08	182	61.69	94	40.69	24	48.00
girls	276	47.92	113	38.31	137	59.31	26	52.00
Consumption of school meals								
yes	210	36.46	100	33.90	89	38.53	21	42.00
no	366	63.54	195	66.10	142	61.47	29	58.00
***Nutritional status***								
Overweight (BAZ ≥ 1) ^§^								
no	291	50.52	82	27.80	164	71.00	45	90.00
yes	285	49.48	213	72.20	67	29.00	5	10.00
Tanner (stage) ^£^								
1–3	327	56.97	172	58.31	121	52.61	34	69.39
4–5	247	43.03	123	41.69	109	47.39	15	30.61
***Sedentary behavior***								
Television watching (h/d) ^£^								
≥2	133	28.18	68	28.22	58	30.37	7	17.50
<2	339	71.82	173	71.78	133	69.63	33	82.50
Sleep time (h/d) ^£^								
≥9	158	30.15	79	29.48	67	31.46	12	27.91
<9	366	69.85	189	70.52	146	68.54	31	72.09
***Maternal characteristics***								
Maternal Obesity ^6,£^								
No	357	63.52	162	56.25	158	70.54	37	74.00
Yes	205	36.48	126	43.75	66	29.46	13	26.00
Highest education level (years) ^£^								
≥12	383	67.67	203	69.76	154	68.14	26	53.06
<12	183	32.33	88	30.24	72	31.86	23	46.94

PR, plausible reporter; UnR, under-reporter; OvR, over-reporter. ^§^ BMI-for-age *z* scores ≥ 1 ^£^ Number missing data: Tanner, 2; sleep time, 52; Television watching, 104; maternal Obesity, 14; highest education level, 10. ^6^ Maternal obesity: BMI ≥ 30 kg/m^2^.

**Table 2:** Odds of being an under-reporter or over-reporter of energy intake compared with being a plausible reporter of energy intake of the participants in the Growth and Obesity Cohort Study 2014–2015.

	UnR			OvR		
	OR	CI 95%		OR	CI 95%	
***Sociodemographic***						
Age, years (mean, SD)	1.64	1.25	2.14	1.22	0.79	1.90
Sex						
boys	1			1		
girls	0.43	0.30	0.61	0.74	0.40	1.37
Consumption of school meals						
yes	1			1		
no	1.22	0.85	1.75	0.87	0.47	1.61
***Nutritional status***						
Overweight (BAZ ≥ 1) ^§^						
no	1			1		
yes	6.36	4.34	9.31	0.27	0.10	0.72
Tanner (stage) ^£^						
1–3	1			1		
4–5	0.79	0.56	1.12	0.49	0.25	0.95
***Sedentary behavior***						
Television watching (h/d) ^£^						
<2	1			1		
≥2	0.90	0.59	1.37	0.49	0.20	1.16
Sleep time (h/d) ^£^						
≥9	1			1		
<9	1.10	0.74	1.62	1.19	0.57	2.45
***Maternal characteristics***						
Maternal Obesity ^6,£^						
No	1			1		
Yes	1.86	1.29	2.69	0.84	0.42	1.68
Highest education level (years) ^£^						
≥12	1			1		
<12	0.93	0.64	1.35	1.89	1.01	3.54
***Nutrient intake***						
Fat (% of total EI)	0.93	0.89	0.96	1.07	1.01	1.13
Carbohydrates (% of total EI)	1.05	1.02	1.09	0.98	0.93	1.03
Proteins (% of total EI)	1.07	0.98	1.15	0.80	0.70	0.91
Saturated Fat (% of total EI)	0.85	0.78	0.93	1.08	0.94	1.23
Sodium mg/1000 kcal	1.00	1.00	1.00	1.00	0.99	1.00
Total Sugar (% of total EI)	1.03	0.94	1.11	1.00	0.94	1.05

UnR, under-reporter; OvR, over-reporter. ^§^ BMI-for-age *z* scores ≥ 1 ^£^ Number missing data: Tanner, 2; sleep time, 52; Television watching, 104; Maternal Obesity, 14; Highest education level, 10. ^6^ Maternal Obesity: BMI ≥ 30 kg/m^2^.

## Data Availability

Data and material can be made available upon request.

## Supplementary Materials

The following supporting information can be downloaded at: https://www.mdpi.com/article/10.3390/children9020293/s1, Table S1: Description of the foods that composed each of the 28 food items included in this study, Chile 2014–2015, Table S2: Reference and cut-off values used to identify misreporting among adolescents, Table S3: Food Items.
