# Supplementary material for: Misreporting of Energy Intake Is Related to Specific Food Items in Low-Middle Income Chilean Adolescents
Source: Children (Basel). 2022 Feb 21;9(2):293. doi: 10.3390/children9020293 (PMC8870629; doi:10.3390/children9020293)
Supplement: Supplementary file 1 [file children-09-00293-s001.zip › children-1541310-supplementary.pdf]

Supplemental Table S1: Description of the foods that composed each of the 29 food groups included in the factor analysis. GOCS Study, Chile 2014-2015.

**Food groups**	**Food composition**
White Milk	Fluid whole milk (3% fat), whole milk powder, reduced fat milk (2% fat), skim milk, skim milk powder
Flavored milk	Mixtures and milk drinks, strawberry, chocolate and other flavors, purchased ready-to-drink
Yogurts	Yogurt, fermented milk
Cheeses	Gouda cheese, ricotta cheese, cream cheese, cheddar cheese,
Meat	Steak beef, ground beef, beef ribs, pork chop, pork ribs, pork loin, poultry, chicken, turkey, fish, viscera (all cooking methods)
Processed meats	Sausage, sausages, frankfurters, meatballs ready for consumption, nuggets, hamburger ready for consumption (only meat), ham, bologna, turkey breast, chicken breast, salami
Junk food	Pizzas, sandwiches of meat or hamburger ready to eat, wonton, egg roll, chilean hot dog, *sopaipillas, tacos, empanadas.* Mainly, street food or fast food
Flavored juice	Juice or flavored drink, purchased ready-to-drink, juice or flavored drink, dry mix - unprepared
Soft drinks	Soda pop or soft drink regular or diet
Bread	French bread, bun bread, white bread with salt
Rice, and pasta	Rice, pasta and dishes pasta.
Potatoes	cooked potatoes, mashed potatoes, , french fries
Vegetables	Lettuce, cabbage, raw salad, others load vegetables. Pumpkin, carrot, cucumber, tomato, among others.
Fruits	Pineapple, banana, orange, apple, pear, papaya, mango, watermelon, tangerine, grape, blueberry, strawberry, blackberry, fruit salad, fruit juices
Eggs	Fried eggs, scrambled eggs, omelet, boiled eggs, egg white, egg yolk
Homemade dishes	Typical chilean food or meals prepared at home, restaurants or schools; that require a longer preparation time and made with natural foods. For instance: vegetables stews with or without meat, *cazuela*, *charquican*, *chapsui*, *humitas, pastel de choclo* among others.
Pulses	Beans, lentils, chickpeas, white beans, legumes-based preparations,
Salty snacks	Crackers, saltine or soda, salty chips – snack type, cheese balls, puffs or twists, potato chips
Chocolates and confectionary	Chocolate candy, chocolate candy bar, sweets based on milk, lollipop, candy, caramel, jams, *dulce de leche*
Sugar	White sugar, honey, brown sugar, syrup, chocolate powder
Ready to eat cereal	Breakfast cereal, oatmeal, whole cereal, granola, corn flakes
Ice cream & popsicles	Ice cream, popsicles, sherbet, dessert frozen
Margarine & butter	Salted butter, unsalted butter, salted margarine, unsalted margarine, light margarine
Desserts	Flan, pudding, Jell-O – gelatin, mousse, canned fruit, chilean desserts
Dipping sauces	Mayonnaise, ketchup, mustard
Oil	Vegetable oil, maravila oil, canola oil
Savory Sauces	Bolognece sauce, bachemel sauce
Cookies & cakes	Cookies and bars, granola bars, sweet biscuit and cookie stuffed, cookie sandwich, cookies commercial packaged, Cakes, cheesecake, cake sponge, doughnut, muffins, pies fruit, cupcake, cake purchased ready-to-eat

Supplemental Table S2: Reference and cut-off values used to identify misreporting among adolescents

	n	CVrEI (%)*	CVpER† (%)	CVmTEE‡ (%)	Plausibility range (% rEI/pER)
**Girls**	276	26.3	5.8	8.2	79.0 to 120.9
**Boys**	300	25.6	5.1	8.2	79.8 to 120.2

CV, coefficient of variation, EI, energy intake; ER, energy requirement; TEE, total energy expenditure.
* Intra-individual variation of EI (24-h recall, GOCS).
†Error in predicted energy expenditure requirements, according to Huang et al and DRI (19, 24).
‡Day-to-day variation and measurement error for TEE based on the double labelled water technique (23).

Supplemental Table S3: Food Items

Food Items	**mean %EI**	**SD**
Bread	17.76	9.24
Rice & Pasta	9.07	7.90
Cookies & Cakes *	7.80	8.25
Junk food	5.99	9.51
Meats	5.35	6.01
Soft Drinks	4.52	4.60
Home dishes	3.65	6.61
Potatoes	3.63	5.44
Processed meats	3.61	4.88
Flavored juice	3.26	3.81
Flavored milk	2.96	4.61
White milk	2.95	4.34
Snacks salt	2.78	5.33
Yogurt	2.66	3.59
Chocolate & confectionery *	2.58	4.17
Fruit	2.46	3.19
Chesses	2.18	3.19
Pulses	2.13	5.00
Vegetables *	2.05	2.52
Sugar	2.00	2.32
Eggs *	1.90	2.89
Ready to eat cereal	1.71	3.13
Ice cream & popcicles	1.18	2.46
Margarine	1.17	1.77
Desserts	1.14	2.99
Dipping sauces	0.92	2.05
Oil	0.80	0.92
Savory sauces	0.79	1.85
